# Lamellar Keratoplasty Combined with Amniotic Membrane Transplantation for the Treatment of Corneal Perforations: A Clinical and In Vivo Confocal Microscopy Study

**DOI:** 10.1155/2020/7403842

**Published:** 2020-02-28

**Authors:** Lan Ke, Dan Shen, Haoyu Wang, Chen Qiao, Qingyan Zeng

**Affiliations:** ^1^Aier Eye Hospital of Wuhan University, Wuhan, Hubei 430063, China; ^2^Hankou Aier Eye Hospital, Wuhan, Hubei 430024, China

## Abstract

**Purpose:**

To evaluate the clinical and in vivo confocal microscopy outcome of lamellar keratoplasty combined with amniotic membrane transplantation for the treatment of corneal perforations.

**Methods:**

In this retrospective, noncomparative, and interventional case series, 13 eyes of 13 patients with corneal perforation were included. All eyes were treated with lamellar keratoplasty combined with amniotic membrane transplantation for corneal reconstruction. Age, underlying etiology, location, size of corneal ulcer, size of corneal perforation, hospitalization days and follow-up time, and corneal confocal microscopy were investigated. Aqueous leakage, anterior chamber formation, epithelial healing time, and visual acuity (VA) were monitored after operation.

**Results:**

The cause of corneal perforation (*n* = 13) was classified as infectious (*n* = 13) was classified as infectious (*n* = 13) was classified as infectious (

**Conclusion:**

Lamellar keratoplasty combined with amniotic membrane transplantation may be an alternative, safe, and effective surgical therapy in the treatment of corneal perforations in the absence of a fresh donor cornea. We recommend this surgery to treat with the size of corneal perforation of <4 mm in diameter no matter peripheral or central corneal perforation, especially who had immune-related diseases.

## 1. Introduction

Corneal perforation is one of the blinding diseases caused by various infectious and noninfectious corneal diseases. It can give rise to irreversible angle-closure glaucoma and even lead to endophthalmitis [1]. Although corneal perforation has a low prevalence in developed countries, it remains one of the major diseases in developing countries that require emergency surgery [2, 3]. In order to maintain the anatomical integrity of the cornea and prevent complications from happening, immediate treatment is required. For the treatment of corneal perforation, we often use surgical and/or nonsurgical methods to intervene. Interventions include wearing soft contact lenses, using tissue biogels [4], simple suturing, conjunctival flap covering surgery, multilayer amniotic membrane transplantation [5, 6], and keratoplasty [7–10].

The choice of treatment option depends on the size, location, and state of the primary disease. At the same time, there are many factors affecting the prognosis of corneal perforation, including the shape of perforation, the presence or absence of iris prolapse, and the degree of anterior chamber hemorrhage. Different surgical treatments also lead to different clinical efficacy [11]. However, wearing soft contact lenses can only temporarily block the smaller aperture perforation; using simple sutures can cause changes in corneal curvature to affect visual acuity (VA); traditional conjunctival flap covering surgery also affect VA and aesthetics; multilayer amniotic membrane transplantation can only temporarily block the perforation of a certain size; although the rejection of lamellar keratoplasty is rare and it does not require fresh corneal donor, it is prone to form double anterior chamber and turbidity of the graft; penetrating keratoplasty is easy to cause immune rejection and affect the recovery of VA. In addition, penetrating keratoplasty requires fresh corneal donors, and the shortage of fresh corneal materials remains a problem in Asian countries, especially in China [12, 13]. But luckily, glycerol-preserved corneas overcome the problem [14]. We considered that glycerol-preserved corneas and amniotic membranes (AMs) may serve as available materials in the treatment of corneal perforations. Herein, we retrospectively reviewed the clinical and in vivo confocal microscopy outcome of treating 13 eyes of 13 patients of corneal perforations by lamellar keratoplasty combined with amniotic membrane transplantation.

## 2. Materials and Methods

### 2.1. Patients

A total of 13 eyes of 13 patients with corneal perforation who were treated with lamellar keratoplasty combined with amniotic membrane transplantation in our hospital from May 2015 to August 2018 were included in our study. All patients could not be cured with a bandage contact lens and had immune factors involved. Patients with corneal perforation due to trauma and purulent corneal ulcer were excluded. The observation included 6 males (6 eyes) and 7 females (7 eyes). The average age was 53.2 ± 14.6 (ranging from 17 to 79) years. This retrospective study was approved by the Ethics Committee of the Hankou Aier Eye Hospital and followed the tenets of the Helsinki Declaration.

### 2.2. Preparation of Grafts and AMs

Preserved human grafts and AMs were obtained from the eye bank of Hankou Aier Eye Hospital. They were all obtained under strict aseptic conditions. The corneal grafts were placed into the prepared sterilized pure glycerin at −20°C for storage. AMs were taken from the placenta of pregnant women who were excluded from potential infectious diseases including AIDS, hepatitis B, hepatitis C, and syphilis. Under sterile conditions, the placenta was thoroughly rinsed 2 to 3 times with the prepared rinse solution. The AM was separated from the placenta, covered on an ethylene oxide-sterilized nitrocellulose membrane, and cut into small pieces of 3 cm × 3 cm size. At last, we placed each AM in a preservation solution at −80°C for use, respectively. Preserved AMs were resuscitated at room temperature for 1 to 2 hours before each use and rehydrated with 0.1% tobramycin solution for 15 minutes.

### 2.3. Surgical Procedures

**Figure 1:**
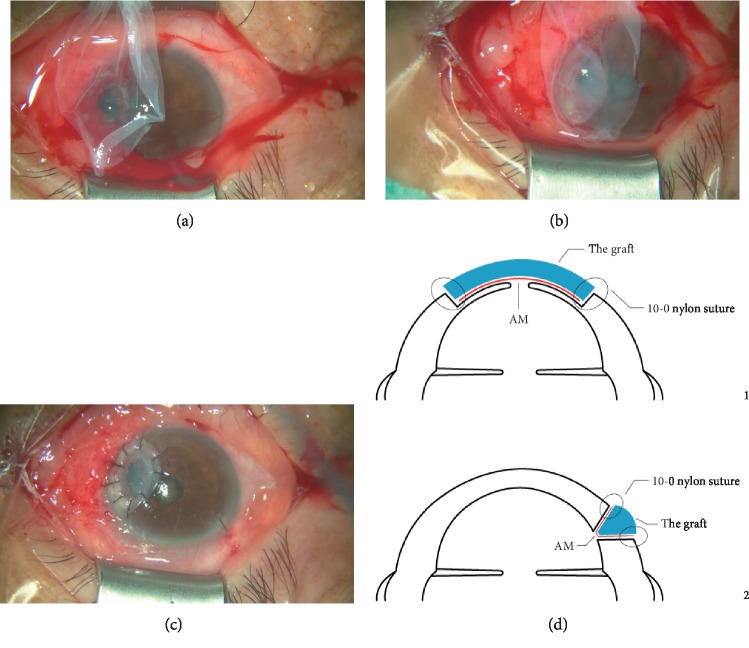
Surgical steps for lamellar keratoplasty combined with amniotic membrane transplantation. (a) The AM was placed flat on the perforation of the cornea. (b) The preserved corneal material was trimmed to a size approximately to the size of the ulcer and then sutured corneal graft over the AM with a 10-0 nylon suture. (c) Trim the excess AM and watertight the anterior chamber. (d) Schematic diagram of the surgical effect (1 was taken when the ulcer was in the center, while 2 was paracentral).

After topical anesthesia and retrobulbar anesthesia, disinfection and surgical drape methods were taken according to the conventional surgical method. The recipient beds were carefully made according to the location and size of the perforation, and then the necrotic tissue and the epithelium covered on the corneal perforation site were completely removed. A lateral corneal incision was made in case of large perforation with iris prolapse. Viscoelastics was injected into the anterior chamber and the perforation site to separate the synechia. At the same time, the preserved AM and corneal graft were taken for resuscitation and rehydration. After the recipient bed was prepared, the AM was placed flat on the perforation of the cornea with the basement membrane side down (Figure 1(a)). The preserved graft was trimmed to a size approximately to the shape of the recipient bed and then sutured it over the AM with a 10-0 nylon suture (Figure 1(b)). Finally, trim the excess AM and watertight the anterior chamber (Figure 1(c)). Bandage contact lens was used until smooth corneal surface was regained. The schematic diagram of the surgical effect is shown in Figure 1(d) (1 was taken when the ulcer was in the center, while 2 was paracentral).

### 2.4. Medical Care after Operation and Follow-Up

All patients were given topical tobramycin dexamethasone ointment, mydriatic, and sodium hyaluronate eye drops four times a day, together with systemic antibiotics and corticosteroids. If the postoperative intraocular pressure was high, oral methazolamide tablets or topical hypotensive drugs may be used selectively. If corneal epithelium repair was observed under the slit-lamp examination, contact lens was taken off and the drug will be adjusted for topical levofloxacin eye drops, prednisolone acetate eye drops (weekly tapering), sodium hyaluronate drops four times a day, and tobramycin dexamethasone ointment once a night. If the primary disease was rheumatoid arthritis, topical 0.1% tacrolimus or 1% cyclosporin A was administered four times a day on the first postoperative day. For herpetic stromal necrotizing keratitis, systemic and topical antiviral drugs were added after surgery. Conjunctival sutures were removed at around one week. All patients were followed up once a week for one month and then once a month after discharge.

### 2.5. Statistics

This study is a retrospective study performed in the Hankou Aier Eye Hospital. The data we reported are presented as the mean ± standard deviation (SD).

## 3. Results

**Figure 2:**
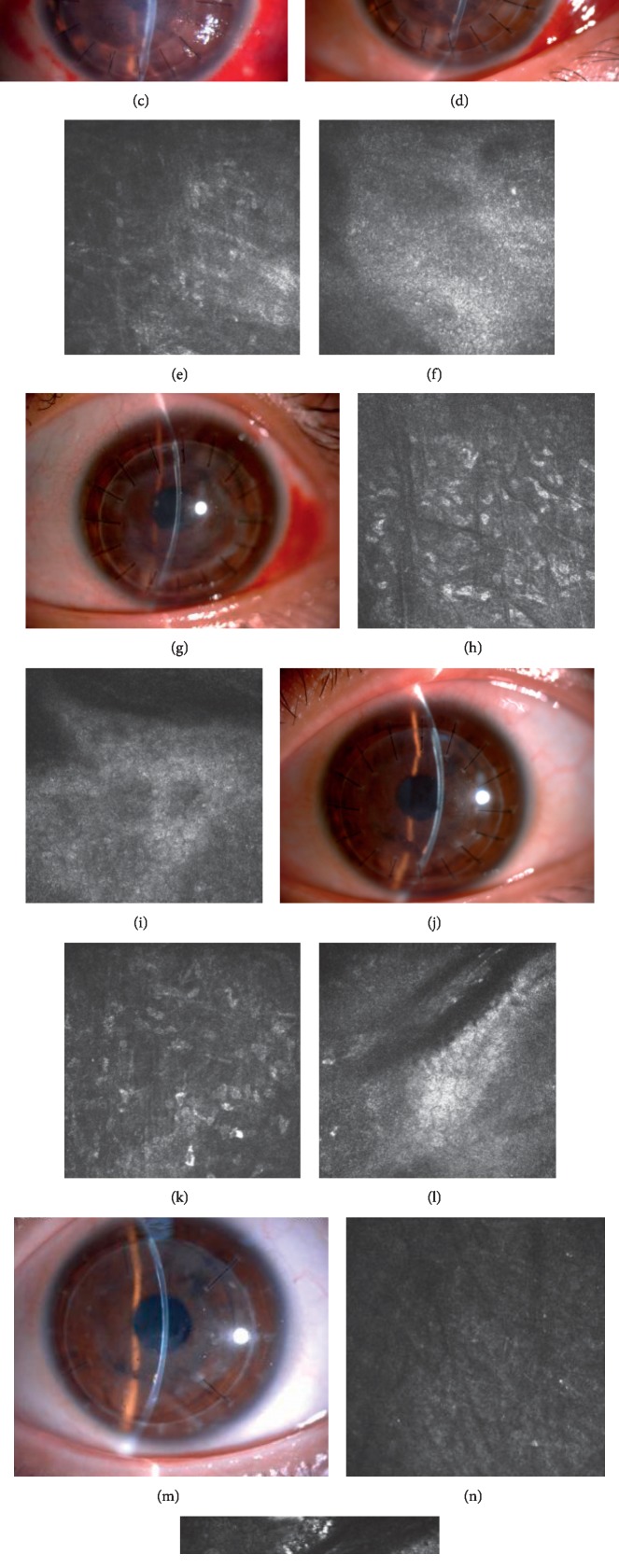
Healing of a central corneal perforation after lamellar keratoplasty combined with amniotic membrane transplantation (Case 1). Corneal perforation was happened during DALK, so we change surgical plan into lamellar keratoplasty combined with amniotic membrane transplantation to remedy. (a) A 17-year-old girl with a corneal ulcer associated with herpetic stromal necrotizing keratitis. The deep ulcer with descemetocele was shown. (b) 3 days after surgery, the anterior chamber was reformed. (c) 8 days after surgery, the corneal edema was reduced. (d) 20 days after surgery, a relatively stable cornea surface with an intrastromal opaque AM layer was seen. (e) Corneal stroma-derived cells were populated in the AM at 20 days after surgery in vivo confocal microscopy images. (f) The structure of the endothelial cell layer was unclear because of corneal edema. (g) 2 months after surgery, a thin demarcation line between the AM and the stroma is visible. (h) Corneal stroma-derived cells were increased at 2 months after surgery in vivo confocal microscopy images. (i) The structure of the endothelial cell layer was unclear at the perforation site and the neighbouring endothelial cells were about 1247 ± 36 mm^2^ at 2 months after surgery in confocal microscopy examination. (j) A nearly normal corneal thickness was seen at 6 months postoperatively, with a low reflective nebula. (k) Corneal stroma-derived cells were still visible with a flaky signal in vivo confocal microscopy images at 6 months postoperatively. (l) Confocal microscopy examination showed the endothelial cell layer at the perforation site which was not smooth. The size of endothelial cells was uniformly increased and the density was about 989 ± 61 mm^2^ at 6 months postoperatively near the perforation site. (m) 20 months after surgery, the corneal surface was totally stable. The VA was better than the preoperative level (from CF to 0.5). (n) Corneal stroma-derived cells were almost not visible in vivo confocal microscopy images. (o) Confocal microscopy examination showed the endothelial cell layer near the perforation site was clear and was about 1143 ± 17 mm^2^ at 20 months after surgery.

Related dates of patients before and after surgery are summarized in Table 1. 13 eyes of 13 patients (6 men and 7 women) with corneal perforation were included. The cause of corneal perforation (*n* = 13) was classified as infectious (*n* = 1) and noninfectious (*n* = 12). Infectious causes included herpetic stromal necrotizing keratitis (*n* = 1). Noninfectious causes included rheumatoid arthritis, Mooren's ulcer, neurotrophic ulcer, severe dry eye, and unknown reason (*n* = 12). Most of the locations of corneal perforation were paracentral, and 2 of them were center. All patients were treated with lamellar keratoplasty combined with amniotic membrane transplantation for corneal reconstruction. All patients received an anatomical cure after surgery. The anterior chambers were formed without aqueous leakage and other complications at postoperative day. One of the patients needed to be discharged from the hospital for systemic radiation therapy, so we were not sure when her corneal epithelium was repaired (Case 6). The mean time of regained a smooth corneal surface was 7.5 ± 2.9 (ranging from 4 to 14) days. The mean hospitalization day was 13.1 ± 4.5 (ranging from 7 to 22) days. The mean follow-up time is 22.5 ± 14.5 (ranging from 4 to 43) months. The AM layers had integrated into the stroma at the perforation ulcer site from the slit-lamp and confocal microscopy (Figure 2). From the slit-lamp microscopy, the AM appeared as a high-reflective reflection in the corneal stroma after surgery about half a year, and at the same time, the high-reflective reflection reduced and the cornea of the perforation ulcer site is almost transparent after surgery about one year, even after one year (Figures 2(a)–2(d), 2(g), 2(j), and 2(m)). From confocal microscopy examination, corneal stroma-derived cells were populated in the AM at about 1 month after surgery, and their density increased at 2 months (Figures 2(e) and 2(h)). Corneal stroma-derived cells were still visible with a flaky signal at 6 months, which were almost not obviously at 20 months postoperatively (Figures 2(k) and 2(n)). The structure of the endothelial cell layer was unclear at early stage. The size of endothelial cells was uniformly increased, and the density was stable after 1 year near the perforation site (Figures 2(f), 2(i), 2(l), and 2(o)). The vision improved to varying degrees in 9 eyes, remained unchanged in 2 eyes, and decreased in 2 eyes. During the follow-up period, one patient underwent lamellar keratoplasty combined with amniotic membrane transplantation again because of the recurrence of the primary disease. No side effects occurred during the follow-up.

## 4. Discussion

The AM is the inner layer of the amnion and the thickest basement membrane of the human body. It consists of three layers: the epithelial layer, the basement membrane layer, and the stromal layer [15, 16]. AMs which promote epithelial growth and inhibit fibrosis, inflammation, and neovascularization have been widely used in ocular surface reconstruction [5, 6, 14, 17–20]. AMs can repair corneal defects as an effective material for corneal reconstruction [5, 6, 14]. Nowadays, the AM has been shown to have anti-inflammatory, antibacterial, antifibrotic, antiangiogenic, and epithelial-promoting effects [10]. Similarly, because of its transparency, lack of immunogenicity, avascularity, and the ability of the corneal and conjunctival epithelial cells to migrate, it is increasingly used in a variety of ocular diseases, such as limbal stem cell deficiency, glaucoma surgery, scleral lysis, bullous keratopathy, and corneal perforation [11].

With the rapid development of ophthalmology, a variety of original surgery using AMs for the treatment of corneal perforation has gradually performed, including the use of ultra-dry-cross-linked AM for surgery [17], “Pleats Fold” technology [18], and “Swiss Roll” technology [19]. Although these techniques all show good results in the literature, there are only good for peripheral corneal perforation, and the use of central corneal perforation requires further surgery, such as penetrating keratoplasty [17–19]. Besides, several clinical studies have reported that the final outcome of patients with corneal ulcers treated with multilayer amniotic membrane transplantation is the formation of a stable avascular leucoma [18, 21–23]. Although the surgery gives a definitive or a temporizing treatment, it still will require keratoplasty.

In our study, we successfully treated 13 eyes of 13 patients with whether peripheral or central corneal perforation with lamellar keratoplasty combined with amniotic membrane transplantation, in which the AM was placed flat under the corneal stroma. The results reported by Namba et al. [18] indicated that the treatment of corneal perforation with AM will eventually result in corneal nebula or corneal macula, and if the optical axis area involves, it will affect vision and eventually need additional surgery. But our results showed that the cornea achieved a certain degree of transparency during follow-up and all patients did not require further surgery. From Figure 2, we can see the AM layers had integrated into the stroma at the perforation ulcer site from the slit-lamp and confocal microscopy. The use of a single layer of AM combined with a preserved transparent lamellar corneal graft can achieve a certain degree of transparency, and we believe that compared to the multilayer AM, the single one has a neatly arranged texture in a certain direction, which can provide a scaffold for corneal stroma-derived cells to migrate and populate, thereby making the keratocytes form a neatly arranged fiber. Although the AM does not possess the same transparency of healthy stroma, it could become more and more transparent as time went by from our observation at the time point of the sixth month or the first year, especially combined with a preserved transparent lamellar corneal graft. It took longer to follow up with these patients to provide us with much more evidence.

Interestingly, no significant aqueous leakage or immune rejection was observed during follow-up in this study. Xie et al. performed penetrating keratoplasty in 52 eyes with corneal perforations secondary to fungal keratitis. Anatomical success could be achieved in all eyes. Postoperative complications such as graft rejection were as high as 38.5%, 12 of which were medically treated and 8 underwent secondary PKP with 4 acquiring clear grafts [24]. Different results may be attributed to the lack of immunogenicity, avascularity, inhibition of inflammation, and neovascularization of the AM. The AM prevents the graft from contacting the aqueous humor, thereby reducing immune rejection and ensuring corneal transparency. Furthermore, the AM can close the gap between the donor cornea and the host bed, thereby improving the tightness of and resist the pressure in the anterior chamber.

From our observation, it is interesting to note that cornea edema and corneal endothelial decompensation did not occur during long-term follow-up. Confocal microscopy is a technique which is helpful to demonstrate the characteristic corneal and conjunctival anatomy at cellular level [21]. So we use it to evaluate the corneal endothelial. From the confocal microscopy examination, the endothelial cells were about 1247 ± 36 mm^2^ at 2 months, 989 ± 61 mm^2^ at 6 months, and 1143 ± 17 mm^2^ at 20 months, respectively. We suspect that there are two reasons for keeping the cornea transparent. One is the cornea in the perforation depends on the pumping function of not only the endothelial cells underneath, but also the peripheral endothelial cells. Another is that the AM may act as the layer of descemet membrane and promote the migration of corneal endothelial cells to maintain the transparency. In the previous study, the application of AMs to ocular diseases is only used to promote corneal epithelial growth and corneal stromal cell migration, and whether the AM can play a role in the corneal endothelium has not been reported in clinic [25]. However, due to the small number of patients and the lack of timely follow-up, the observation of the role of AMs in the host cornea at cellular level was limited. Therefore, we are not able to give sufficient evidence for our speculation. In addition, we are going to establish animal models to observe the role of AMs in perforating cornea. It is hoped that it can have a good reference value for the clinical work of lamellar keratoplasty combined with amniotic membrane transplantation for the treatment of corneal perforation.

According to rigorous research, our study should set up experimental and control groups, such as undergoing sole lamellar keratoplasty or amniotic membrane transplantation, to further validate the effect of our surgery. However, we had difficulty establishing a control group because most of the patients who participated in the study had immune-related diseases and had a long course of disease. In addition, there are limitations in the number of patients with corneal perforation and long postoperative recovery. Despite these limitations, we have found that lamellar keratoplasty combined with amniotic membrane transplantation may prove to be good treatment options for whether peripheral or central corneal perforation.

In summary, lamellar keratoplasty combined with amniotic membrane transplantation is an effective and safe treatment alternative to other surgery in the absence of a fresh donor cornea. Based on the results of our long-term follow-up, lamellar keratoplasty combined with amniotic membrane transplantation maintains the integrity of the anatomy of the cornea and some patients recovered part of their vision and did not induce corneal neovascularization. Unnecessary trauma to the donor site can be avoided as compared to traditional conjunctival flap covering surgery; otherwise, it is cosmetically acceptable. It has a lower probability of forming a double anterior chamber and maintains the transparency of the cornea compared to lamellar keratoplasty. In addition, this surgery does not require fresh donor cornea compared to penetrating keratoplasty. Since most of our patients had immune-related diseases and the perforation sites were located paracentral with dry eye, lamellar keratoplasty had much more advantages than PKP with long follow-up stability. Therefore, we recommend this surgery to treat with the size of corneal perforation of <4 mm in diameter no matter peripheral or central corneal perforation, especially who had immune-related diseases. However, there is still a need for long-term studies of larger samples of patients with various sizes of perforations.

## Figures and Tables

**Table 1:** Related dates of patients before and after improved DALK.

Case	Age (yrs)	Sex	Eye	Underlying etiology	Location	Size of corneal ulcer (mm^2^)	Size of corneal perforation (mm^2^)	VA		Aqueous leakage	Anterior chamber formation	Epithelial healing time	Hospitalization days	Follow-up (mos)
								Before	After					
1	17	F	OS	Herpetic stromal necrotizing keratitis	Center	3 × 4	1 × 1	CF	0.5	No	Yes	6	11	18
2	57	F	OS	Rheumatoid arthritis	Paracentral	1 × 2	1 × 1	0.02	0.2	No	Yes	11	15	24
3	58	F	OD	Rheumatoid arthritis	Paracentral	2 × 4	0.5 × 1	0.25	0.3	No	Yes	5	10	41
4	56	F	OS	Mooren's ulcer	Paracentral	3 × 3	1 × 1	0.2	0.2	No	Yes	6	9	30
5	68	F	OS	Rheumatoid arthritis	Paracentral	2 × 4	1 × 3	HM	CF	No	Yes	10	22	15
6	38	F	OS	Neurotrophic ulcer	Center	2 × 2.5	1.5 × 1.5	HM	HM	No	Yes	-	8	8
7	52	F	OS	Severe dry eye	Paracentral	3 × 4	1 × 2	HM	0.12	No	Yes	10	12	5
8	55	M	OD	Mooren's ulcer	Paracentral	2.5 × 8	1.5 × 2	0.04	0.02	No	Yes	5	14	5
9	79	M	OD	Rheumatoid arthritis	Paracentral	2.5 × 4.5	2 × 4	0.1	0.06	No	Yes	4	7	4
10	63	M	OD	Unknown reason	Paracentral	1.5 × 3	0.8 × 1	HM	0.3	No	Yes	6	13	18
11	59	M	OD	Mooren's ulcer	Paracentral	6 × 11	1 × 1	HM	0.1	No	Yes	6	22	43
12	40	M	OD	Mooren's ulcer	Paracentral	3 × 6	1 × 2	0.2	0.3	No	Yes	7	12	39
13	50	M	OD	Mooren's ulcer	Paracentral	1.5 × 9	1 × 1	0.12	0.5	No	Yes	14	16	42

OD = oculus dexter; OS = oculus sinister; CF = counting fingers; HM = hand motion; VA = visual acuity; mos = months; yrs = years.
